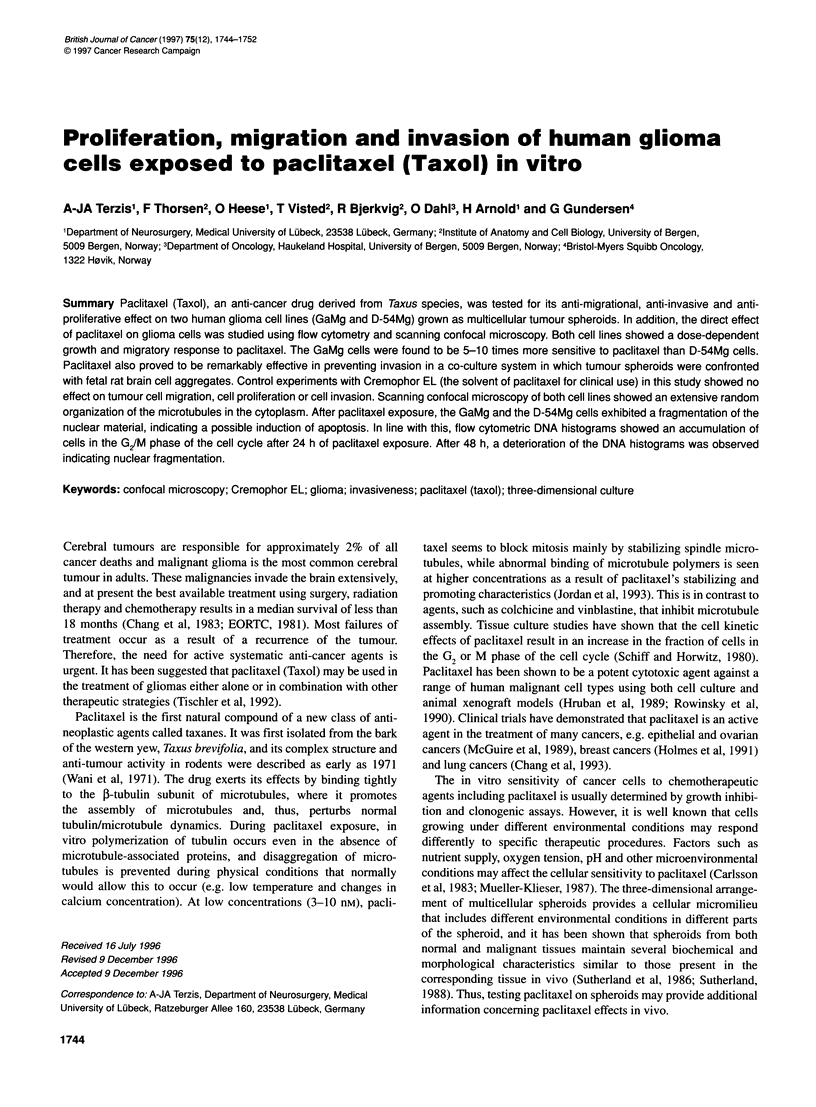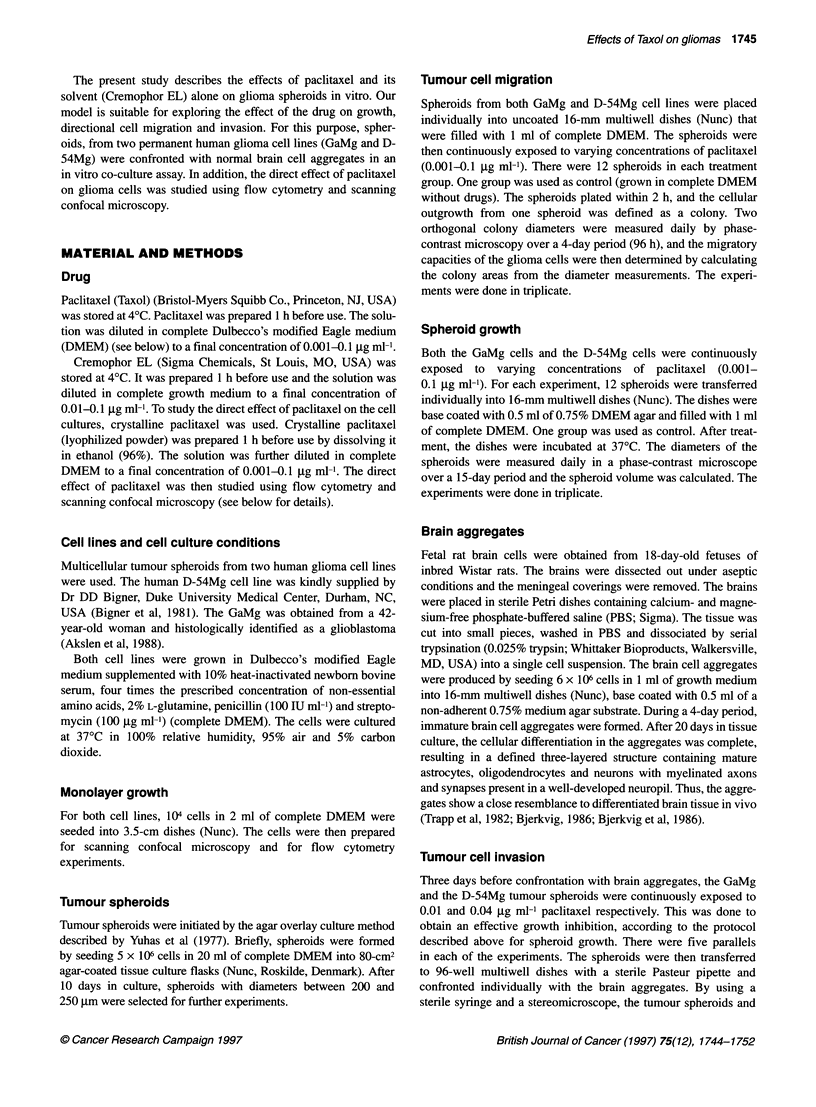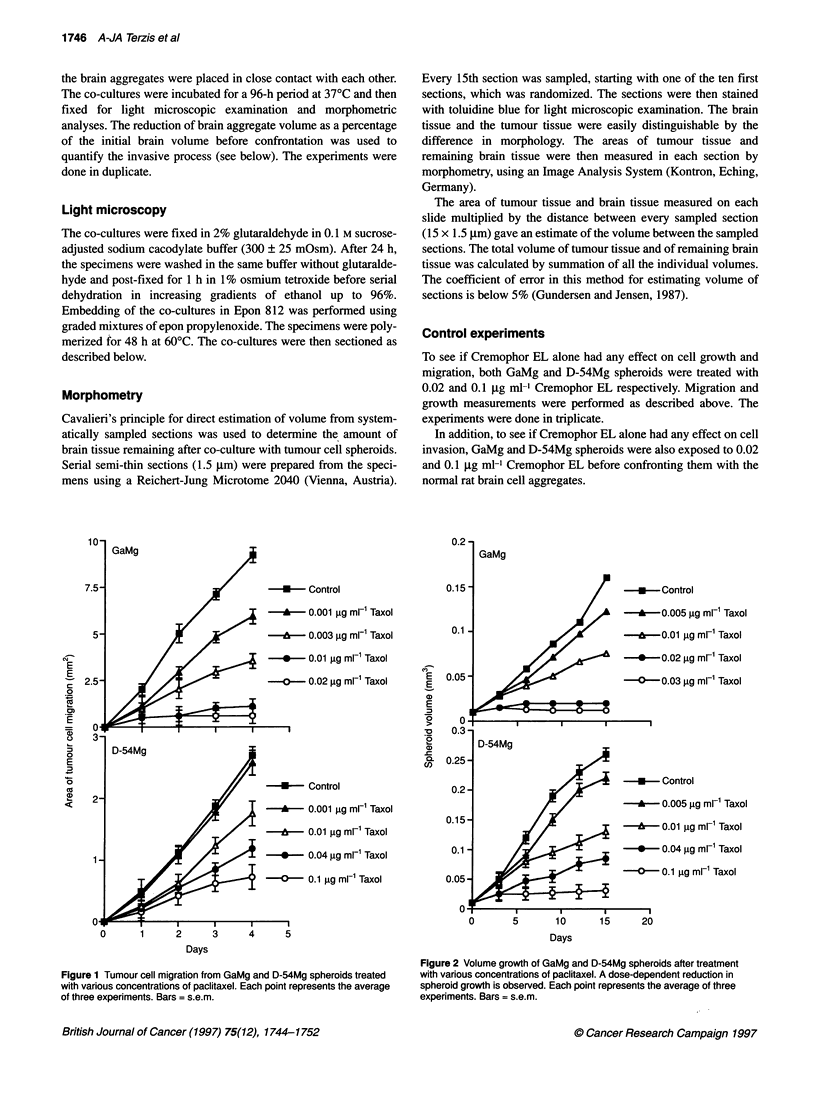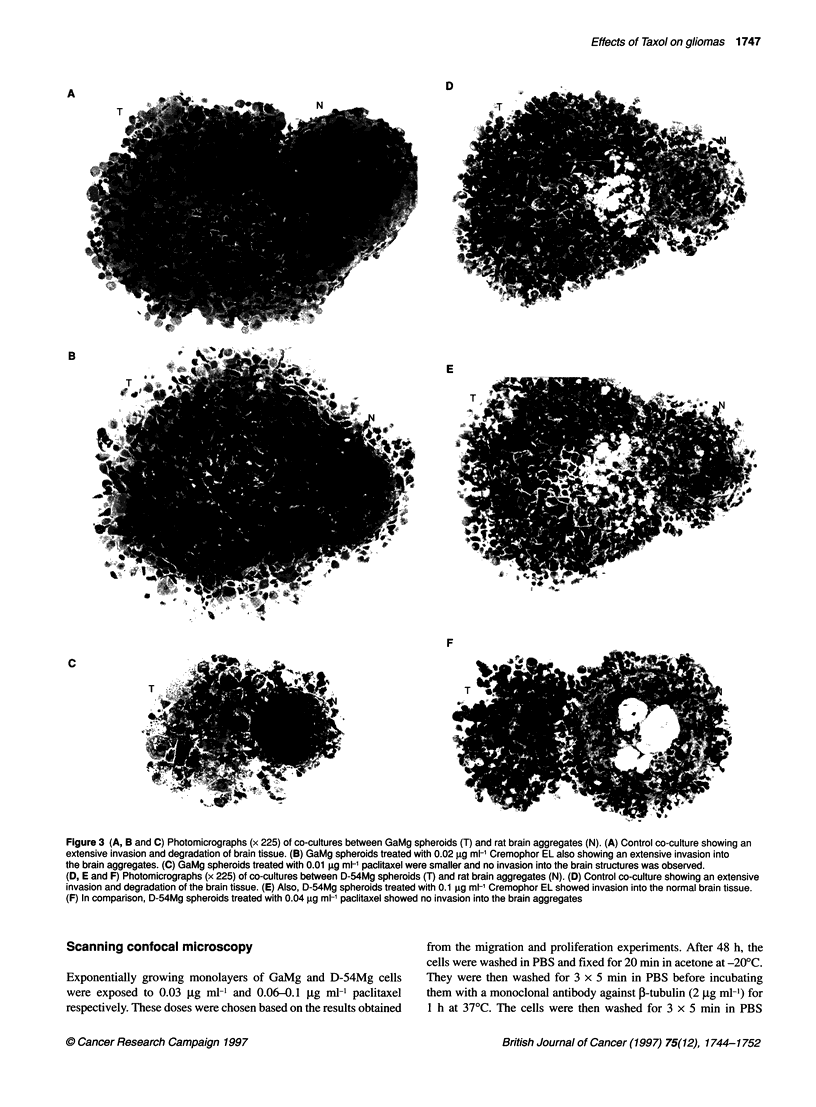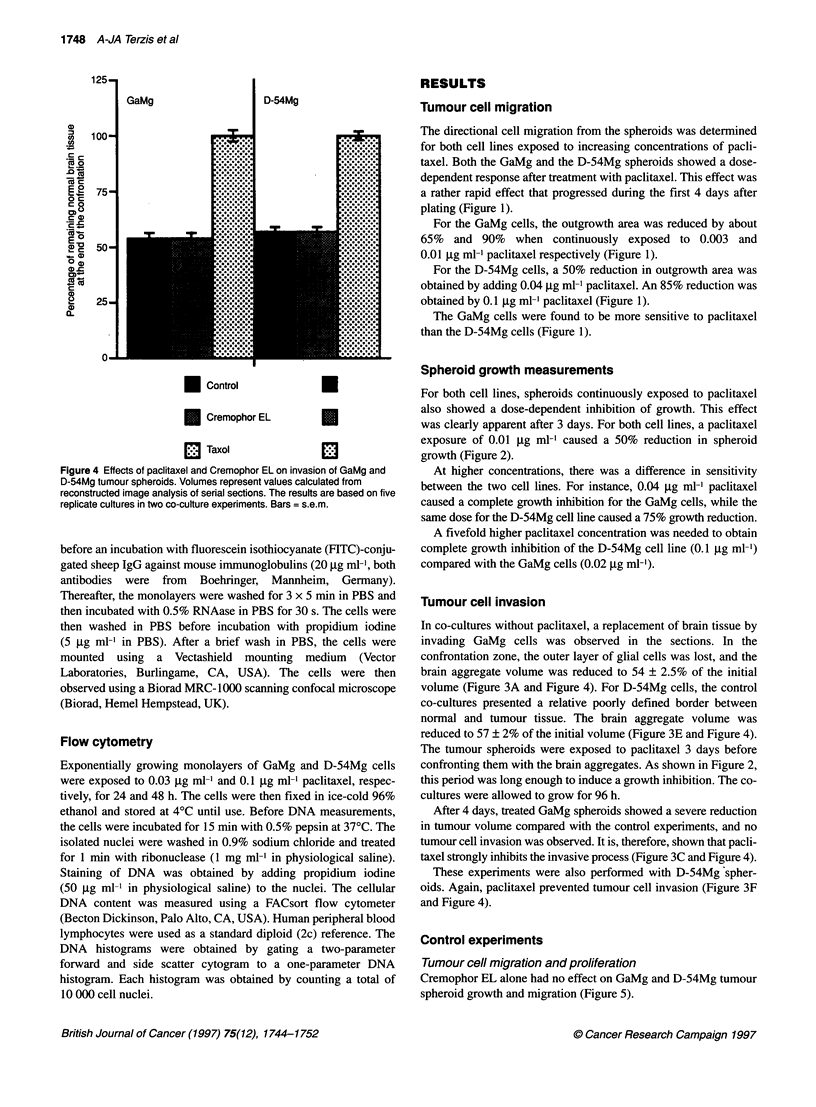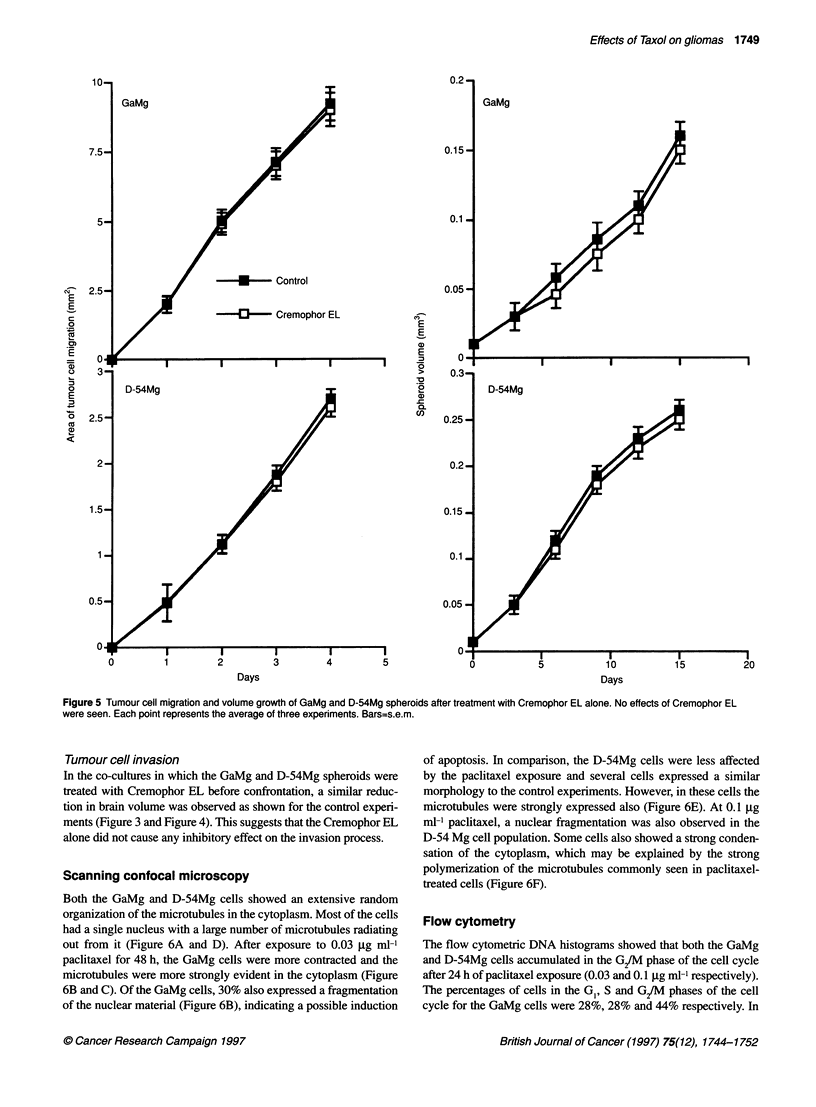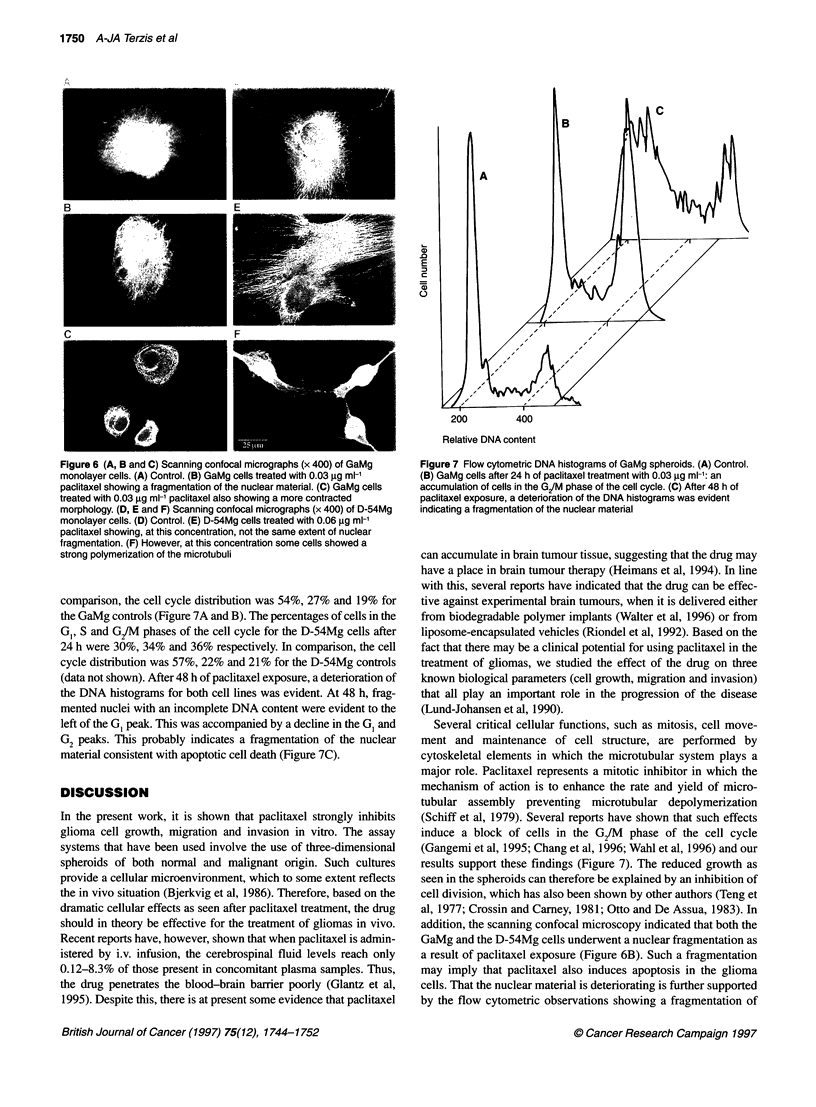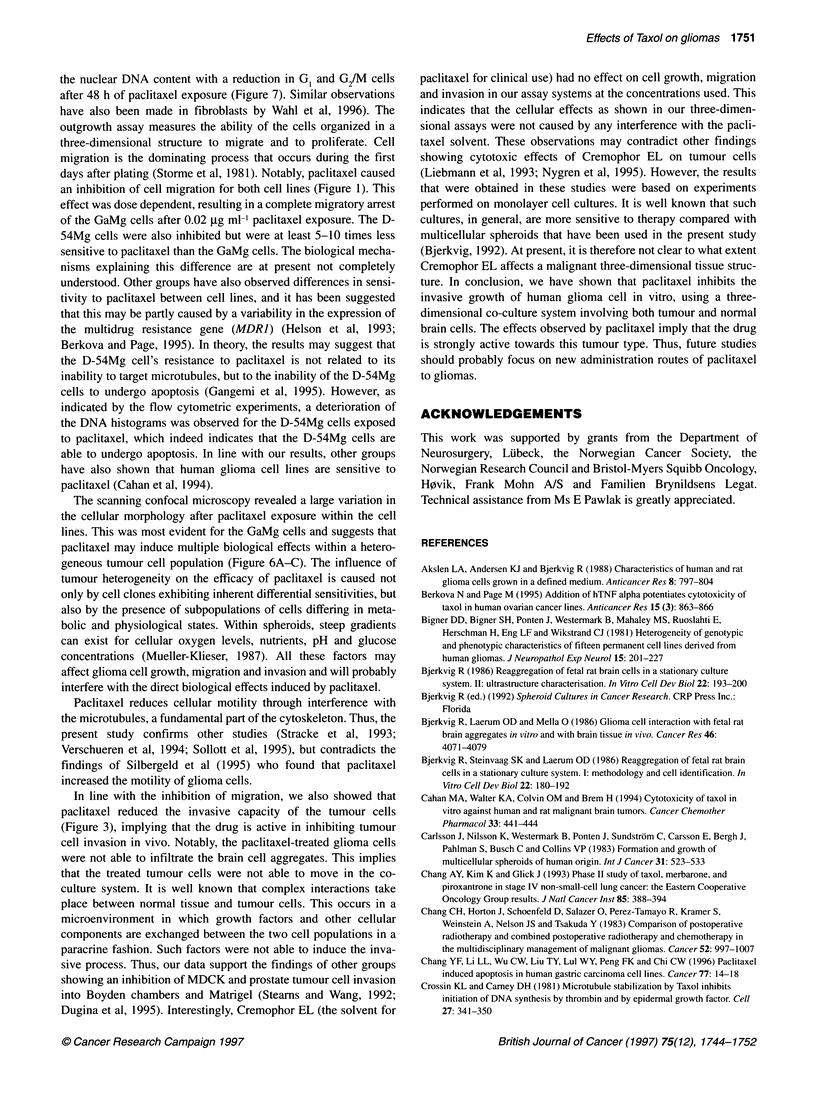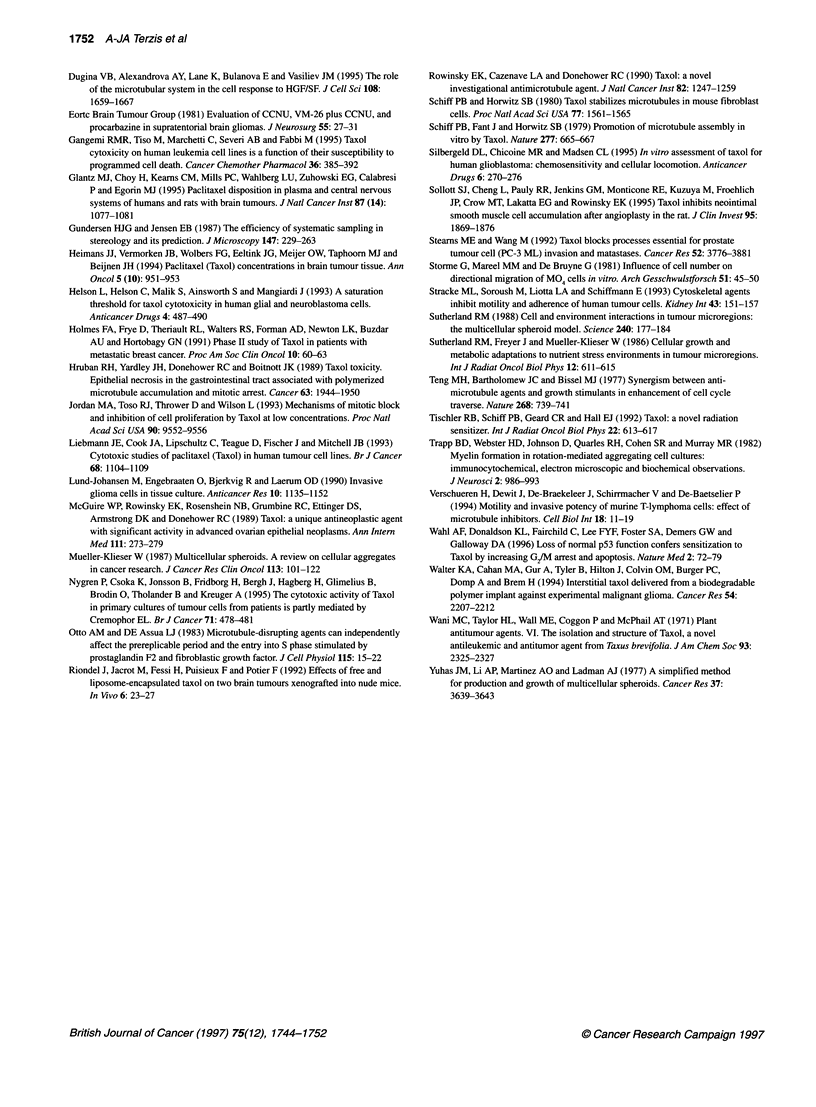# Proliferation, migration and invasion of human glioma cells exposed to paclitaxel (Taxol) in vitro.

**DOI:** 10.1038/bjc.1997.298

**Published:** 1997

**Authors:** A. J. Terzis, F. Thorsen, O. Heese, T. Visted, R. Bjerkvig, O. Dahl, H. Arnold, G. Gundersen

**Affiliations:** Department of Neurosurgery, Medical University of LÃ¼beck, Germany.

## Abstract

**Images:**


British Journal of Cancer (1997) 75(12), 1744–1752
© 1997 Cancer Research Campaign

### Proliferation, migration and invasion of human glioma cells exposed to paclitaxel (Taxol) in vitro

A-JA Terzis[1], F Thorsen[2], O Heese[1], T Visted[2], R Bjerkvig[2], O Dahl[3], H Arnold[1] and G Gundersen[4]

[1]Department of Neurosurgery, Medical University of Lübeck, 23538 Lübeck, Germany; [2]Institute of Anatomy and Cell Biology, University of Bergen, 5009 Bergen, Norway; [3]Department of Oncology, Haukeland Hospital, University of Bergen, 5009 Bergen, Norway; [4]Bristol-Myers Squibb Oncology, 1322 Høvik, Norway

**Summary** Paclitaxel (Taxol), an anti-cancer drug derived from *Taxus* species, was tested for its anti-migrational, anti-invasive and anti-proliferative effect on two human glioma cell lines (GaMg and D-54Mg) grown as multicellular tumour spheroids. In addition, the direct effect of paclitaxel on glioma cells was studied using flow cytometry and scanning confocal microscopy. Both cell lines showed a dose-dependent growth and migratory response to paclitaxel. The GaMg cells were found to be 5–10 times more sensitive to paclitaxel than D-54Mg cells. Paclitaxel also proved to be remarkably effective in preventing invasion in a co-culture system in which tumour spheroids were confronted with fetal rat brain cell aggregates. Control experiments with Cremophor EL (the solvent of paclitaxel for clinical use) in this study showed no effect on tumour cell migration, cell proliferation or cell invasion. Scanning confocal microscopy of both cell lines showed an extensive random organization of the microtubules in the cytoplasm. After paclitaxel exposure, the GaMg and the D-54Mg cells exhibited a fragmentation of the nuclear material, indicating a possible induction of apoptosis. In line with this, flow cytometric DNA histograms showed an accumulation of cells in the $G_2$/M phase of the cell cycle after 24 h of paclitaxel exposure. After 48 h, a deterioration of the DNA histograms was observed indicating nuclear fragmentation.

**Keywords:** confocal microscopy; Cremophor EL; glioma; invasiveness; paclitaxel (taxol); three-dimensional culture

Cerebral tumours are responsible for approximately 2% of all cancer deaths and malignant glioma is the most common cerebral tumour in adults. These malignancies invade the brain extensively, and at present the best available treatment using surgery, radiation therapy and chemotherapy results in a median survival of less than 18 months (Chang et al, 1983; EORTC, 1981). Most failures of treatment occur as a result of a recurrence of the tumour. Therefore, the need for active systematic anti-cancer agents is urgent. It has been suggested that paclitaxel (Taxol) may be used in the treatment of gliomas either alone or in combination with other therapeutic strategies (Tischler et al, 1992).

Paclitaxel is the first natural compound of a new class of anti-neoplastic agents called taxanes. It was first isolated from the bark of the western yew, *Taxus brevifolia*, and its complex structure and anti-tumour activity in rodents were described as early as 1971 (Wani et al, 1971). The drug exerts its effects by binding tightly to the β-tubulin subunit of microtubules, where it promotes the assembly of microtubules and, thus, perturbs normal tubulin/microtubule dynamics. During paclitaxel exposure, in vitro polymerization of tubulin occurs even in the absence of microtubule-associated proteins, and disaggregation of microtubules is prevented during physical conditions that normally would allow this to occur (e.g. low temperature and changes in calcium concentration). At low concentrations (3–10 nM), paclitaxel seems to block mitosis mainly by stabilizing spindle microtubules, while abnormal binding of microtubule polymers is seen at higher concentrations as a result of paclitaxel's stabilizing and promoting characteristics (Jordan et al, 1993). This is in contrast to agents, such as colchicine and vinblastine, that inhibit microtubule assembly. Tissue culture studies have shown that the cell kinetic effects of paclitaxel result in an increase in the fraction of cells in the $G_2$ or M phase of the cell cycle (Schiff and Horwitz, 1980). Paclitaxel has been shown to be a potent cytotoxic agent against a range of human malignant cell types using both cell culture and animal xenograft models (Hruban et al, 1989; Rowinsky et al, 1990). Clinical trials have demonstrated that paclitaxel is an active agent in the treatment of many cancers, e.g. epithelial and ovarian cancers (McGuire et al, 1989), breast cancers (Holmes et al, 1991) and lung cancers (Chang et al, 1993).

The in vitro sensitivity of cancer cells to chemotherapeutic agents including paclitaxel is usually determined by growth inhibition and clonogenic assays. However, it is well known that cells growing under different environmental conditions may respond differently to specific therapeutic procedures. Factors such as nutrient supply, oxygen tension, pH and other microenvironmental conditions may affect the cellular sensitivity to paclitaxel (Carlsson et al, 1983; Mueller-Klieser, 1987). The three-dimensional arrangement of multicellular spheroids provides a cellular micromilieu that includes different environmental conditions in different parts of the spheroid, and it has been shown that spheroids from both normal and malignant tissues maintain several biochemical and morphological characteristics similar to those present in the corresponding tissue in vivo (Sutherland et al, 1986; Sutherland, 1988). Thus, testing paclitaxel on spheroids may provide additional information concerning paclitaxel effects in vivo.

*Received 16 July 1996*
*Revised 9 December 1996*
*Accepted 9 December 1996*

*Correspondence to:* A-JA Terzis, Department of Neurosurgery, Medical University of Lübeck, Ratzeburger Allee 160, 23538 Lübeck, Germany

The present study describes the effects of paclitaxel and its solvent (Cremophor EL) alone on glioma spheroids in vitro. Our model is suitable for exploring the effect of the drug on growth, directional cell migration and invasion. For this purpose, spheroids, from two permanent human glioma cell lines (GaMg and D-54Mg) were confronted with normal brain cell aggregates in an in vitro co-culture assay. In addition, the direct effect of paclitaxel on glioma cells was studied using flow cytometry and scanning confocal microscopy.

### MATERIAL AND METHODS

### Drug

Paclitaxel (Taxol) (Bristol-Myers Squibb Co., Princeton, NJ, USA) was stored at 4°C. Paclitaxel was prepared 1 h before use. The solution was diluted in complete Dulbecco's modified Eagle medium (DMEM) (see below) to a final concentration of 0.001–0.1 μg $ml^{-1}$.

Cremophor EL (Sigma Chemicals, St Louis, MO, USA) was stored at 4°C. It was prepared 1 h before use and the solution was diluted in complete growth medium to a final concentration of 0.01–0.1 μg $ml^{-1}$. To study the direct effect of paclitaxel on the cell cultures, crystalline paclitaxel was used. Crystalline paclitaxel (lyophilized powder) was prepared 1 h before use by dissolving it in ethanol (96%). The solution was further diluted in complete DMEM to a final concentration of 0.001–0.1 μg $ml^{-1}$. The direct effect of paclitaxel was then studied using flow cytometry and scanning confocal microscopy (see below for details).

### Cell lines and cell culture conditions

Multicellular tumour spheroids from two human glioma cell lines were used. The human D-54Mg cell line was kindly supplied by Dr DD Bigner, Duke University Medical Center, Durham, NC, USA (Bigner et al, 1981). The GaMg was obtained from a 42-year-old woman and histologically identified as a glioblastoma (Akslen et al, 1988).

Both cell lines were grown in Dulbecco's modified Eagle medium supplemented with 10% heat-inactivated newborn bovine serum, four times the prescribed concentration of non-essential amino acids, 2% L-glutamine, penicillin (100 IU $ml^{-1}$) and streptomycin (100 μg $ml^{-1}$) (complete DMEM). The cells were cultured at 37°C in 100% relative humidity, 95% air and 5% carbon dioxide.

### Monolayer growth

For both cell lines, $10^4$ cells in 2 ml of complete DMEM were seeded into 3.5-cm dishes (Nunc). The cells were then prepared for scanning confocal microscopy and for flow cytometry experiments.

### Tumour spheroids

Tumour spheroids were initiated by the agar overlay culture method described by Yuhas et al (1977). Briefly, spheroids were formed by seeding $5 \times 10^6$ cells in 20 ml of complete DMEM into 80-$cm^2$ agar-coated tissue culture flasks (Nunc, Roskilde, Denmark). After 10 days in culture, spheroids with diameters between 200 and 250 μm were selected for further experiments.

### Tumour cell migration

Spheroids from both GaMg and D-54Mg cell lines were placed individually into uncoated 16-mm multiwell dishes (Nunc) that were filled with 1 ml of complete DMEM. The spheroids were then continuously exposed to varying concentrations of paclitaxel (0.001–0.1 μg $ml^{-1}$). There were 12 spheroids in each treatment group. One group was used as control (grown in complete DMEM without drugs). The spheroids plated within 2 h, and the cellular outgrowth from one spheroid was defined as a colony. Two orthogonal colony diameters were measured daily by phase-contrast microscopy over a 4-day period (96 h), and the migratory capacities of the glioma cells were then determined by calculating the colony areas from the diameter measurements. The experiments were done in triplicate.

### Spheroid growth

Both the GaMg cells and the D-54Mg cells were continuously exposed to varying concentrations of paclitaxel (0.001–0.1 μg $ml^{-1}$). For each experiment, 12 spheroids were transferred individually into 16-mm multiwell dishes (Nunc). The dishes were base coated with 0.5 ml of 0.75% DMEM agar and filled with 1 ml of complete DMEM. One group was used as control. After treatment, the dishes were incubated at 37°C. The diameters of the spheroids were measured daily in a phase-contrast microscope over a 15-day period and the spheroid volume was calculated. The experiments were done in triplicate.

### Brain aggregates

Fetal rat brain cells were obtained from 18-day-old fetuses of inbred Wistar rats. The brains were dissected out under aseptic conditions and the meningeal coverings were removed. The brains were placed in sterile Petri dishes containing calcium- and magnesium-free phosphate-buffered saline (PBS; Sigma). The tissue was cut into small pieces, washed in PBS and dissociated by serial trypsination (0.025% trypsin; Whittaker Bioproducts, Walkersville, MD, USA) into a single cell suspension. The brain cell aggregates were produced by seeding $6 \times 10^6$ cells in 1 ml of growth medium into 16-mm multiwell dishes (Nunc), base coated with 0.5 ml of a non-adherent 0.75% medium agar substrate. During a 4-day period, immature brain cell aggregates were formed. After 20 days in tissue culture, the cellular differentiation in the aggregates was complete, resulting in a defined three-layered structure containing mature astrocytes, oligodendrocytes and neurons with myelinated axons and synapses present in a well-developed neuropil. Thus, the aggregates show a close resemblance to differentiated brain tissue in vivo (Trapp et al, 1982; Bjerkvig, 1986; Bjerkvig et al, 1986).

### Tumour cell invasion

Three days before confrontation with brain aggregates, the GaMg and the D-54Mg tumour spheroids were continuously exposed to 0.01 and 0.04 μg $ml^{-1}$ paclitaxel respectively. This was done to obtain an effective growth inhibition, according to the protocol described above for spheroid growth. There were five parallels in each of the experiments. The spheroids were then transferred to 96-well multiwell dishes with a sterile Pasteur pipette and confronted individually with the brain aggregates. By using a sterile syringe and a stereomicroscope, the tumour spheroids and

© Cancer Research Campaign 1997

the brain aggregates were placed in close contact with each other. The co-cultures were incubated for a 96-h period at 37°C and then fixed for light microscopic examination and morphometric analyses. The reduction of brain aggregate volume as a percentage of the initial brain volume before confrontation was used to quantify the invasive process (see below). The experiments were done in duplicate.

### Light microscopy

The co-cultures were fixed in 2% glutaraldehyde in 0.1 M sucrose-adjusted sodium cacodylate buffer (300 ± 25 mOsm). After 24 h, the specimens were washed in the same buffer without glutaraldehyde and post-fixed for 1 h in 1% osmium tetroxide before serial dehydration in increasing gradients of ethanol up to 96%. Embedding of the co-cultures in Epon 812 was performed using graded mixtures of epon propylenoxide. The specimens were polymerized for 48 h at 60°C. The co-cultures were then sectioned as described below.

### Morphometry

Cavalieri's principle for direct estimation of volume from systematically sampled sections was used to determine the amount of brain tissue remaining after co-culture with tumour cell spheroids. Serial semi-thin sections (1.5 μm) were prepared from the specimens using a Reichert-Jung Microtome 2040 (Vienna, Austria). Every 15th section was sampled, starting with one of the ten first sections, which was randomized. The sections were then stained with toluidine blue for light microscopic examination. The brain tissue and the tumour tissue were easily distinguishable by the difference in morphology. The areas of tumour tissue and remaining brain tissue were then measured in each section by morphometry, using an Image Analysis System (Kontron, Eching, Germany).

The area of tumour tissue and brain tissue measured on each slide multiplied by the distance between every sampled section (15 × 1.5 μm) gave an estimate of the volume between the sampled sections. The total volume of tumour tissue and of remaining brain tissue was calculated by summation of all the individual volumes. The coefficient of error in this method for estimating volume of sections is below 5% (Gundersen and Jensen, 1987).

### Control experiments

To see if Cremophor EL alone had any effect on cell growth and migration, both GaMg and D-54Mg spheroids were treated with 0.02 and 0.1 μg $ml^{-1}$ Cremophor EL respectively. Migration and growth measurements were performed as described above. The experiments were done in triplicate.

In addition, to see if Cremophor EL alone had any effect on cell invasion, GaMg and D-54Mg spheroids were also exposed to 0.02 and 0.1 μg $ml^{-1}$ Cremophor EL before confronting them with the normal rat brain cell aggregates.

**Figure 1** Tumour cell migration from GaMg and D-54Mg spheroids treated with various concentrations of paclitaxel. Each point represents the average of three experiments. Bars = s.e.m.

**Figure 2** Volume growth of GaMg and D-54Mg spheroids after treatment with various concentrations of paclitaxel. A dose-dependent reduction in spheroid growth is observed. Each point represents the average of three experiments. Bars = s.e.m.

© Cancer Research Campaign 1997

**Figure 3** (**A, B** and **C**) Photomicrographs (× 225) of co-cultures between GaMg spheroids (T) and rat brain aggregates (N). (**A**) Control co-culture showing an extensive invasion and degradation of brain tissue. (**B**) GaMg spheroids treated with 0.02 μg ml$^{-1}$ Cremophor EL also showing an extensive invasion into the brain aggregates. (**C**) GaMg spheroids treated with 0.01 μg ml$^{-1}$ paclitaxel were smaller and no invasion into the brain structures was observed. (**D, E** and **F**) Photomicrographs (× 225) of co-cultures between D-54Mg spheroids (T) and rat brain aggregates (N). (**D**) Control co-culture showing an extensive invasion and degradation of the brain tissue. (**E**) Also, D-54Mg spheroids treated with 0.1 μg ml$^{-1}$ Cremophor EL showed invasion into the normal brain tissue. (**F**) In comparison, D-54Mg spheroids treated with 0.04 μg ml$^{-1}$ paclitaxel showed no invasion into the brain aggregates

### Scanning confocal microscopy

Exponentially growing monolayers of GaMg and D-54Mg cells were exposed to 0.03 μg ml$^{-1}$ and 0.06–0.1 μg ml$^{-1}$ paclitaxel respectively. These doses were chosen based on the results obtained from the migration and proliferation experiments. After 48 h, the cells were washed in PBS and fixed for 20 min in acetone at –20°C. They were then washed for 3 × 5 min in PBS before incubating them with a monoclonal antibody against β-tubulin (2 μg ml$^{-1}$) for 1 h at 37°C. The cells were then washed for 3 × 5 min in PBS

*© Cancer Research Campaign 1997*

**Figure 4** Effects of paclitaxel and Cremophor EL on invasion of GaMg and D-54Mg tumour spheroids. Volumes represent values calculated from reconstructed image analysis of serial sections. The results are based on five replicate cultures in two co-culture experiments. Bars = s.e.m.

before an incubation with fluorescein isothiocyanate (FITC)-conjugated sheep IgG against mouse immunoglobulins (20 μg ml$^{-1}$, both antibodies were from Boehringer, Mannheim, Germany). Thereafter, the monolayers were washed for 3 × 5 min in PBS and then incubated with 0.5% RNAase in PBS for 30 s. The cells were then washed in PBS before incubation with propidium iodine (5 μg ml$^{-1}$ in PBS). After a brief wash in PBS, the cells were mounted using a Vectashield mounting medium (Vector Laboratories, Burlingame, CA, USA). The cells were then observed using a Biorad MRC-1000 scanning confocal microscope (Biorad, Hemel Hempstead, UK).

### Flow cytometry

Exponentially growing monolayers of GaMg and D-54Mg cells were exposed to 0.03 μg ml$^{-1}$ and 0.1 μg ml$^{-1}$ paclitaxel, respectively, for 24 and 48 h. The cells were then fixed in ice-cold 96% ethanol and stored at 4°C until use. Before DNA measurements, the cells were incubated for 15 min with 0.5% pepsin at 37°C. The isolated nuclei were washed in 0.9% sodium chloride and treated for 1 min with ribonuclease (1 mg ml$^{-1}$ in physiological saline). Staining of DNA was obtained by adding propidium iodine (50 μg ml$^{-1}$ in physiological saline) to the nuclei. The cellular DNA content was measured using a FACsort flow cytometer (Becton Dickinson, Palo Alto, CA, USA). Human peripheral blood lymphocytes were used as a standard diploid (2c) reference. The DNA histograms were obtained by gating a two-parameter forward and side scatter cytogram to a one-parameter DNA histogram. Each histogram was obtained by counting a total of 10 000 cell nuclei.

### RESULTS

### Tumour cell migration

The directional cell migration from the spheroids was determined for both cell lines exposed to increasing concentrations of paclitaxel. Both the GaMg and the D-54Mg spheroids showed a dose-dependent response after treatment with paclitaxel. This effect was a rather rapid effect that progressed during the first 4 days after plating (Figure 1).

For the GaMg cells, the outgrowth area was reduced by about 65% and 90% when continuously exposed to 0.003 and 0.01 μg ml$^{-1}$ paclitaxel respectively (Figure 1).

For the D-54Mg cells, a 50% reduction in outgrowth area was obtained by adding 0.04 μg ml$^{-1}$ paclitaxel. An 85% reduction was obtained by 0.1 μg ml$^{-1}$ paclitaxel (Figure 1).

The GaMg cells were found to be more sensitive to paclitaxel than the D-54Mg cells (Figure 1).

### Spheroid growth measurements

For both cell lines, spheroids continuously exposed to paclitaxel also showed a dose-dependent inhibition of growth. This effect was clearly apparent after 3 days. For both cell lines, a paclitaxel exposure of 0.01 μg ml$^{-1}$ caused a 50% reduction in spheroid growth (Figure 2).

At higher concentrations, there was a difference in sensitivity between the two cell lines. For instance, 0.04 μg ml$^{-1}$ paclitaxel caused a complete growth inhibition for the GaMg cells, while the same dose for the D-54Mg cell line caused a 75% growth reduction.

A fivefold higher paclitaxel concentration was needed to obtain complete growth inhibition of the D-54Mg cell line (0.1 μg ml$^{-1}$) compared with the GaMg cells (0.02 μg ml$^{-1}$).

### Tumour cell invasion

In co-cultures without paclitaxel, a replacement of brain tissue by invading GaMg cells was observed in the sections. In the confrontation zone, the outer layer of glial cells was lost, and the brain aggregate volume was reduced to 54 ± 2.5% of the initial volume (Figure 3A and Figure 4). For D-54Mg cells, the control co-cultures presented a relative poorly defined border between normal and tumour tissue. The brain aggregate volume was reduced to 57 ± 2% of the initial volume (Figure 3E and Figure 4). The tumour spheroids were exposed to paclitaxel 3 days before confronting them with the brain aggregates. As shown in Figure 2, this period was long enough to induce a growth inhibition. The co-cultures were allowed to grow for 96 h.

After 4 days, treated GaMg spheroids showed a severe reduction in tumour volume compared with the control experiments, and no tumour cell invasion was observed. It is, therefore, shown that paclitaxel strongly inhibits the invasive process (Figure 3C and Figure 4).

These experiments were also performed with D-54Mg spheroids. Again, paclitaxel prevented tumour cell invasion (Figure 3F and Figure 4).

### Control experiments

#### *Tumour cell migration and proliferation*

Cremophor EL alone had no effect on GaMg and D-54Mg tumour spheroid growth and migration (Figure 5).

 © Cancer Research Campaign 1997

**Figure 5** Tumour cell migration and volume growth of GaMg and D-54Mg spheroids after treatment with Cremophor EL alone. No effects of Cremophor EL were seen. Each point represents the average of three experiments. Bars=s.e.m.

### *Tumour cell invasion*

In the co-cultures in which the GaMg and D-54Mg spheroids were treated with Cremophor EL before confrontation, a similar reduction in brain volume was observed as shown for the control experiments (Figure 3 and Figure 4). This suggests that the Cremophor EL alone did not cause any inhibitory effect on the invasion process.

### Scanning confocal microscopy

Both the GaMg and D-54Mg cells showed an extensive random organization of the microtubules in the cytoplasm. Most of the cells had a single nucleus with a large number of microtubules radiating out from it (Figure 6A and D). After exposure to 0.03 μg ml$^{-1}$ paclitaxel for 48 h, the GaMg cells were more contracted and the microtubules were more strongly evident in the cytoplasm (Figure 6B and C). Of the GaMg cells, 30% also expressed a fragmentation of the nuclear material (Figure 6B), indicating a possible induction of apoptosis. In comparison, the D-54Mg cells were less affected by the paclitaxel exposure and several cells expressed a similar morphology to the control experiments. However, in these cells the microtubules were strongly expressed also (Figure 6E). At 0.1 μg ml$^{-1}$ paclitaxel, a nuclear fragmentation was also observed in the D-54 Mg cell population. Some cells also showed a strong condensation of the cytoplasm, which may be explained by the strong polymerization of the microtubules commonly seen in paclitaxel-treated cells (Figure 6F).

### Flow cytometry

The flow cytometric DNA histograms showed that both the GaMg and D-54Mg cells accumulated in the $G_2/M$ phase of the cell cycle after 24 h of paclitaxel exposure (0.03 and 0.1 μg ml$^{-1}$ respectively). The percentages of cells in the $G_1$, S and $G_2/M$ phases of the cell cycle for the GaMg cells were 28%, 28% and 44% respectively. In

© Cancer Research Campaign 1997

Figure 6 (A, B and C) Scanning confocal micrographs (× 400) of GaMg monolayer cells. (A) Control. (B) GaMg cells treated with 0.03 μg $ml^{-1}$ paclitaxel showing a fragmentation of the nuclear material. (C) GaMg cells treated with 0.03 μg $ml^{-1}$ paclitaxel also showing a more contracted morphology. (D, E and F) Scanning confocal micrographs (× 400) of D-54Mg monolayer cells. (D) Control. (E) D-54Mg cells treated with 0.06 μg $ml^{-1}$ paclitaxel showing, at this concentration, not the same extent of nuclear fragmentation. (F) However, at this concentration some cells showed a strong polymerization of the microtubuli

Figure 7 Flow cytometric DNA histograms of GaMg spheroids. (A) Control. (B) GaMg cells after 24 h of paclitaxel treatment with 0.03 μg $ml^{-1}$: an accumulation of cells in the $G_2$/M phase of the cell cycle. (C) After 48 h of paclitaxel exposure, a deterioration of the DNA histograms was evident indicating a fragmentation of the nuclear material comparison, the cell cycle distribution was 54%, 27% and 19% for the GaMg controls (Figure 7A and B). The percentages of cells in the $G_1$, S and $G_2$/M phases of the cell cycle for the D-54Mg cells after 24 h were 30%, 34% and 36% respectively. In comparison, the cell cycle distribution was 57%, 22% and 21% for the D-54Mg controls (data not shown). After 48 h of paclitaxel exposure, a deterioration of the DNA histograms for both cell lines was evident. At 48 h, fragmented nuclei with an incomplete DNA content were evident to the left of the $G_1$ peak. This was accompanied by a decline in the $G_1$ and $G_2$ peaks. This probably indicates a fragmentation of the nuclear material consistent with apoptotic cell death (Figure 7C).

### DISCUSSION

In the present work, it is shown that paclitaxel strongly inhibits glioma cell growth, migration and invasion in vitro. The assay systems that have been used involve the use of three-dimensional spheroids of both normal and malignant origin. Such cultures provide a cellular microenvironment, which to some extent reflects the in vivo situation (Bjerkvig et al, 1986). Therefore, based on the dramatic cellular effects as seen after paclitaxel treatment, the drug should in theory be effective for the treatment of gliomas in vivo. Recent reports have, however, shown that when paclitaxel is administered by i.v. infusion, the cerebrospinal fluid levels reach only 0.12–8.3% of those present in concomitant plasma samples. Thus, the drug penetrates the blood–brain barrier poorly (Glantz et al, 1995). Despite this, there is at present some evidence that paclitaxel can accumulate in brain tumour tissue, suggesting that the drug may have a place in brain tumour therapy (Heimans et al, 1994). In line with this, several reports have indicated that the drug can be effective against experimental brain tumours, when it is delivered either from biodegradable polymer implants (Walter et al, 1996) or from liposome-encapsulated vehicles (Riondel et al, 1992). Based on the fact that there may be a clinical potential for using paclitaxel in the treatment of gliomas, we studied the effect of the drug on three known biological parameters (cell growth, migration and invasion) that all play an important role in the progression of the disease (Lund-Johansen et al, 1990).

Several critical cellular functions, such as mitosis, cell movement and maintenance of cell structure, are performed by cytoskeletal elements in which the microtubular system plays a major role. Paclitaxel represents a mitotic inhibitor in which the mechanism of action is to enhance the rate and yield of microtubular assembly preventing microtubular depolymerization (Schiff et al, 1979). Several reports have shown that such effects induce a block of cells in the $G_2$/M phase of the cell cycle (Gangemi et al, 1995; Chang et al, 1996; Wahl et al, 1996) and our results support these findings (Figure 7). The reduced growth as seen in the spheroids can therefore be explained by an inhibition of cell division, which has also been shown by other authors (Teng et al, 1977; Crossin and Carney, 1981; Otto and De Assua, 1983). In addition, the scanning confocal microscopy indicated that both the GaMg and the D-54Mg cells underwent a nuclear fragmentation as a result of paclitaxel exposure (Figure 6B). Such a fragmentation may imply that paclitaxel also induces apoptosis in the glioma cells. That the nuclear material is deteriorating is further supported by the flow cytometric observations showing a fragmentation of

© Cancer Research Campaign 1997

the nuclear DNA content with a reduction in $G_1$ and $G_2/M$ cells after 48 h of paclitaxel exposure (Figure 7). Similar observations have also been made in fibroblasts by Wahl et al, 1996). The outgrowth assay measures the ability of the cells organized in a three-dimensional structure to migrate and to proliferate. Cell migration is the dominating process that occurs during the first days after plating (Storme et al, 1981). Notably, paclitaxel caused an inhibition of cell migration for both cell lines (Figure 1). This effect was dose dependent, resulting in a complete migratory arrest of the GaMg cells after 0.02 $\mu$g ml$^{-1}$ paclitaxel exposure. The D-54Mg cells were also inhibited but were at least 5–10 times less sensitive to paclitaxel than the GaMg cells. The biological mechanisms explaining this difference are at present not completely understood. Other groups have also observed differences in sensitivity to paclitaxel between cell lines, and it has been suggested that this may be partly caused by a variability in the expression of the multidrug resistance gene (*MDR1*) (Helson et al, 1993; Berkova and Page, 1995). In theory, the results may suggest that the D-54Mg cell's resistance to paclitaxel is not related to its inability to target microtubules, but to the inability of the D-54Mg cells to undergo apoptosis (Gangemi et al, 1995). However, as indicated by the flow cytometric experiments, a deterioration of the DNA histograms was observed for the D-54Mg cells exposed to paclitaxel, which indeed indicates that the D-54Mg cells are able to undergo apoptosis. In line with our results, other groups have also shown that human glioma cell lines are sensitive to paclitaxel (Cahan et al, 1994).

The scanning confocal microscopy revealed a large variation in the cellular morphology after paclitaxel exposure within the cell lines. This was most evident for the GaMg cells and suggests that paclitaxel may induce multiple biological effects within a heterogeneous tumour cell population (Figure 6A–C). The influence of tumour heterogeneity on the efficacy of paclitaxel is caused not only by cell clones exhibiting inherent differential sensitivities, but also by the presence of subpopulations of cells differing in metabolic and physiological states. Within spheroids, steep gradients can exist for cellular oxygen levels, nutrients, pH and glucose concentrations (Mueller-Klieser, 1987). All these factors may affect glioma cell growth, migration and invasion and will probably interfere with the direct biological effects induced by paclitaxel.

Paclitaxel reduces cellular motility through interference with the microtubules, a fundamental part of the cytoskeleton. Thus, the present study confirms other studies (Stracke et al, 1993; Verschueren et al, 1994; Sollott et al, 1995), but contradicts the findings of Silbergeld et al (1995) who found that paclitaxel increased the motility of glioma cells.

In line with the inhibition of migration, we also showed that paclitaxel reduced the invasive capacity of the tumour cells (Figure 3), implying that the drug is active in inhibiting tumour cell invasion in vivo. Notably, the paclitaxel-treated glioma cells were not able to infiltrate the brain cell aggregates. This implies that the treated tumour cells were not able to move in the co-culture system. It is well known that complex interactions take place between normal tissue and tumour cells. This occurs in a microenvironment in which growth factors and other cellular components are exchanged between the two cell populations in a paracrine fashion. Such factors were not able to induce the invasive process. Thus, our data support the findings of other groups showing an inhibition of MDCK and prostate tumour cell invasion into Boyden chambers and Matrigel (Stearns and Wang, 1992; Dugina et al, 1995). Interestingly, Cremophor EL (the solvent for paclitaxel for clinical use) had no effect on cell growth, migration and invasion in our assay systems at the concentrations used. This indicates that the cellular effects as shown in our three-dimensional assays were not caused by any interference with the paclitaxel solvent. These observations may contradict other findings showing cytotoxic effects of Cremophor EL on tumour cells (Liebmann et al, 1993; Nygren et al, 1995). However, the results that were obtained in these studies were based on experiments performed on monolayer cell cultures. It is well known that such cultures, in general, are more sensitive to therapy compared with multicellular spheroids that have been used in the present study (Bjerkvig, 1992). At present, it is therefore not clear to what extent Cremophor EL affects a malignant three-dimensional tissue structure. In conclusion, we have shown that paclitaxel inhibits the invasive growth of human glioma cell in vitro, using a three-dimensional co-culture system involving both tumour and normal brain cells. The effects observed by paclitaxel imply that the drug is strongly active towards this tumour type. Thus, future studies should probably focus on new administration routes of paclitaxel to gliomas.

### ACKNOWLEDGEMENTS

This work was supported by grants from the Department of Neurosurgery, Lübeck, the Norwegian Cancer Society, the Norwegian Research Council and Bristol-Myers Squibb Oncology, Høvik, Frank Mohn A/S and Familien Brynildsens Legat. Technical assistance from Ms E Pawlak is greatly appreciated.

### REFERENCES

Akslen LA, Andersen KJ and Bjerkvig R (1988) Characteristics of human and rat glioma cells grown in a defined medium. *Anticancer Res* **8**: 797–804

Berkova N and Page M (1995) Addition of hTNF alpha potentiates cytotoxicity of taxol in human ovarian cancer lines. *Anticancer Res* **15** (**3**): 863–866

Bigner DD, Bigner SH, Ponten J, Westermark B, Mahaley MS, Ruoslahti E, Herschman H, Eng LF and Wikstrand CJ (1981) Heterogeneity of genotypic and phenotypic characteristics of fifteen permanent cell lines derived from human gliomas. *J Neuropathol Exp Neurol* **15**: 201–227

Bjerkvig R (1986) Reaggregation of fetal rat brain cells in a stationary culture system. II: ultrastructure characterisation. *In Vitro Cell Dev Biol* **22**: 193–200

Bjerkvig R (ed.) (1992) *Spheroid Cultures in Cancer Research.* CRP Press Inc.: Florida

Bjerkvig R, Laerum OD and Mella O (1986) Glioma cell interaction with fetal rat brain aggregates *in vitro* and with brain tissue *in vivo. Cancer Res* **46**: 4071–4079

Bjerkvig R, Steinvaag SK and Laerum OD (1986) Reaggregation of fetal rat brain cells in a stationary culture system. I: methodology and cell identification. *In Vitro Cell Dev Biol* **22**: 180–192

Cahan MA, Walter KA, Colvin OM and Brem H (1994) Cytotoxicity of taxol in vitro against human and rat malignant brain tumors. *Cancer Chemother Pharmacol* **33**: 441–444

Carlsson J, Nilsson K, Westermark B, Ponten J, Sundström C, Carsson E, Bergh J, Pahlman S, Busch C and Collins VP (1983) Formation and growth of multicellular spheroids of human origin. *Int J Cancer* **31**: 523–533

Chang AY, Kim K and Glick J (1993) Phase II study of taxol, merbarone, and piroxantrone in stage IV non-small-cell lung cancer: the Eastern Cooperative Oncology Group results. *J Natl Cancer Inst* **85**: 388–394

Chang CH, Horton J, Schoenfeld D, Salazer O, Perez-Tamayo R, Kramer S, Weinstein A, Nelson JS and Tsakuda Y (1983) Comparison of postoperative radiotherapy and combined postoperative radiotherapy and chemotherapy in the multidisciplinary management of malignant gliomas. *Cancer* **52**: 997–1007

Chang YF, Li LL, Wu CW, Liu TY, Lul WY, Peng FK and Chi CW (1996) Paclitaxel induced apoptosis in human gastric carcinoma cell lines. *Cancer* **77**: 14–18

Crossin KL and Carney DH (1981) Microtubule stabilization by Taxol inhibits initiation of DNA synthesis by thrombin and by epidermal growth factor. *Cell* **27**: 341–350

© Cancer Research Campaign 1997 

Dugina VB, Alexandrova AY, Lane K, Bulanova E and Vasiliev JM (1995) The role of the microtubular system in the cell response to HGF/SF. *J Cell Sci* **108**: 1659–1667

Eortc Brain Tumour Group (1981) Evaluation of CCNU, VM-26 plus CCNU, and procarbazine in supratentorial brain gliomas. *J Neurosurg* **55**: 27–31

Gangemi RMR, Tiso M, Marchetti C, Severi AB and Fabbi M (1995) Taxol cytoxicity on human leukemia cell lines is a function of their susceptibility to programmed cell death. *Cancer Chemother Pharmacol* **36**: 385–392

Glantz MJ, Choy H, Kearns CM, Mills PC, Wahlberg LU, Zuhowski EG, Calabresi P and Egorin MJ (1995) Paclitaxel disposition in plasma and central nervous systems of humans and rats with brain tumours. *J Natl Cancer Inst* **87 (14)**: 1077–1081

Gundersen HJG and Jensen EB (1987) The efficiency of systematic sampling in stereology and its prediction. *J Microscopy* **147**: 229–263

Heimans JJ, Vermorken JB, Wolbers FG, Eeltink JG, Meijer OW, Taphoorn MJ and Beijnen JH (1994) Paclitaxel (Taxol) concentrations in brain tumour tissue. *Ann Oncol* **5 (10)**: 951–953

Helson L, Helson C, Malik S, Ainsworth S and Mangiardi J (1993) A saturation threshold for taxol cytotoxicity in human glial and neuroblastoma cells. *Anticancer Drugs* **4**: 487–490

Holmes FA, Frye D, Theriault RL, Walters RS, Forman AD, Newton LK, Buzdar AU and Hortobagy GN (1991) Phase II study of Taxol in patients with metastatic breast cancer. *Proc Am Soc Clin Oncol* **10**: 60–63

Hruban RH, Yardley JH, Donehower RC and Boitnott JK (1989) Taxol toxicity. Epithelial necrosis in the gastrointestinal tract associated with polymerized microtubule accumulation and mitotic arrest. *Cancer* **63**: 1944–1950

Jordan MA, Toso RJ, Thrower D and Wilson L (1993) Mechanisms of mitotic block and inhibition of cell proliferation by Taxol at low concentrations. *Proc Natl Acad Sci USA* **90**: 9552–9556

Liebmann JE, Cook JA, Lipschultz C, Teague D, Fischer J and Mitchell JB (1993) Cytotoxic studies of paclitaxel (Taxol) in human tumour cell lines. *Br J Cancer* **68**: 1104–1109

Lund-Johansen M, Engebraaten O, Bjerkvig R and Laerum OD (1990) Invasive glioma cells in tissue culture. *Anticancer Res* **10**: 1135–1152

McGuire WP, Rowinsky EK, Rosenshein NB, Grumbine RC, Ettinger DS, Armstrong DK and Donehower RC (1989) Taxol: a unique antineoplastic agent with significant activity in advanced ovarian epithelial neoplasms. *Ann Intern Med* **111**: 273–279

Mueller-Klieser W (1987) Multicellular spheroids. A review on cellular aggregates in cancer research. *J Cancer Res Clin Oncol* **113**: 101–122

Nygren P, Csoka K, Jonsson B, Fridborg H, Bergh J, Hagberg H, Glimelius B, Brodin O, Tholander B and Kreuger A (1995) The cytotoxic activity of Taxol in primary cultures of tumour cells from patients is partly mediated by Cremophor EL. *Br J Cancer* **71**: 478–481

Otto AM and DE Assua LJ (1983) Microtubule-disrupting agents can independently affect the prereplicable period and the entry into S phase stimulated by prostaglandin F2 and fibroblastic growth factor. *J Cell Physiol* **115**: 15–22

Riondel J, Jacrot M, Fessi H, Puisieux F and Potier F (1992) Effects of free and liposome-encapsulated taxol on two brain tumours xenografted into nude mice. *In Vivo* **6**: 23–27

Rowinsky EK, Cazenave LA and Donehower RC (1990) Taxol: a novel investigational antimicrotubule agent. *J Natl Cancer Inst* **82**: 1247–1259

Schiff PB and Horwitz SB (1980) Taxol stabilizes microtubules in mouse fibroblast cells. *Proc Natl Acad Sci USA* **77**: 1561–1565

Schiff PB, Fant J and Horwitz SB (1979) Promotion of microtubule assembly in vitro by Taxol. *Nature* **277**: 665–667

Silbergeld DL, Chicoine MR and Madsen CL (1995) *In vitro* assessment of taxol for human glioblastoma: chemosensitivity and cellular locomotion. *Anticancer Drugs* **6**: 270–276

Sollott SJ, Cheng L, Pauly RR, Jenkins GM, Monticone RE, Kuzuya M, Froehlich JP, Crow MT, Lakatta EG and Rowinsky EK (1995) Taxol inhibits neointimal smooth muscle cell accumulation after angioplasty in the rat. *J Clin Invest* **95**: 1869–1876

Stearns ME and Wang M (1992) Taxol blocks processes essential for prostate tumour cell (PC-3 ML) invasion and matastases. *Cancer Res* **52**: 3776–3881

Storme G, Mareel MM and De Bruyne G (1981) Influence of cell number on directional migration of $MO_4$ cells *in vitro*. *Arch Gesschwulstforsch* **51**: 45–50

Stracke ML, Soroush M, Liotta LA and Schiffmann E (1993) Cytoskeletal agents inhibit motility and adherence of human tumour cells. *Kidney Int* **43**: 151–157

Sutherland RM (1988) Cell and environment interactions in tumour microregions: the multicellular spheroid model. *Science* **240**: 177–184

Sutherland RM, Freyer J and Mueller-Klieser W (1986) Cellular growth and metabolic adaptations to nutrient stress environments in tumour microregions. *Int J Radiat Oncol Biol Phys* **12**: 611–615

Teng MH, Bartholomew JC and Bissel MJ (1977) Synergism between anti-microtubule agents and growth stimulants in enhancement of cell cycle traverse. *Nature* **268**: 739–741

Tischler RB, Schiff PB, Geard CR and Hall EJ (1992) Taxol: a novel radiation sensitizer. *Int J Radiat Oncol Biol Phys* **22**: 613–617

Trapp BD, Webster HD, Johnson D, Quarles RH, Cohen SR and Murray MR (1982) Myelin formation in rotation-mediated aggregating cell cultures: immunocytochemical, electron microscopic and biochemical observations. *J Neurosci* **2**: 986–993

Verschueren H, Dewit J, De-Braekeleer J, Schirrmacher V and De-Baetselier P (1994) Motility and invasive potency of murine T-lymphoma cells: effect of microtubule inhibitors. *Cell Biol Int* **18**: 11–19

Wahl AF, Donaldson KL, Fairchild C, Lee FYF, Foster SA, Demers GW and Galloway DA (1996) Loss of normal p53 function confers sensitization to Taxol by increasing $G_2/M$ arrest and apoptosis. *Nature Med* **2**: 72–79

Walter KA, Cahan MA, Gur A, Tyler B, Hilton J, Colvin OM, Burger PC, Domp A and Brem H (1994) Interstitial taxol delivered from a biodegradable polymer implant against experimental malignant glioma. *Cancer Res* **54**: 2207–2212

Wani MC, Taylor HL, Wall ME, Coggon P and McPhail AT (1971) Plant antitumour agents. VI. The isolation and structure of Taxol, a novel antileukemic and antitumor agent from *Taxus brevifolia*. *J Am Chem Soc* **93**: 2325–2327

Yuhas JM, Li AP, Martinez AO and Ladman AJ (1977) A simplified method for production and growth of multicellular spheroids. *Cancer Res* **37**: 3639–3643

© Cancer Research Campaign 1997